# Effect of reaction solvent on hydroxyapatite synthesis in sol–gel process

**DOI:** 10.1098/rsos.171098

**Published:** 2017-12-20

**Authors:** Muhammad Anwaar Nazeer, Emel Yilgor, Mustafa Baris Yagci, Ugur Unal, Iskender Yilgor

**Affiliations:** Kuytam Surface Science and Technology Center, Chemistry Department, Koç University, Istanbul, Turkey

**Keywords:** hydroxyapatite, β-calcium pyrophosphate, biphasic calcium phosphate, sol–gel process, bone tissue engineering

## Abstract

Synthesis of hydroxyapatite (HA) through sol–gel process in different solvent systems is reported. Calcium nitrate tetrahydrate (CNTH) and diammonium hydrogen phosphate (DAHP) were used as calcium and phosphorus precursors, respectively. Three different synthesis reactions were carried out by changing the solvent media, while keeping all other process parameters constant. A measure of 0.5 M aqueous DAHP solution was used in all reactions while CNTH was dissolved in distilled water, tetrahydrofuran (THF) and *N*,*N*-dimethylformamide (DMF) at a concentration of 0.5 M. Ammonia solution (28–30%) was used to maintain the pH of the reaction mixtures in the 10–12 range. All reactions were carried out at 40 ± 2°C for 4 h. Upon completion of the reactions, products were filtered, washed and calcined at 500°C for 2 h. It was clearly demonstrated through various techniques that the dielectric constant and polarity of the solvent mixture strongly influence the chemical structure and morphological properties of calcium phosphate synthesized. Water-based reaction medium, with highest dielectric constant, mainly produced β-calcium pyrophosphate (β-CPF) with a minor amount of HA. DMF/water system yielded HA as the major phase with a very minor amount of β-CPF. THF/water solvent system with the lowest dielectric constant resulted in the formation of pure HA.

## 1. Introduction

Hydroxyapatite [Ca_5_(PO_4_)_3_OH] (HA), also denoted as [Ca_10_(PO_4_)_6_(OH)_2_] in order to show the hexagonal crystal unit cell consisting of two entities, is an important and extensively studied material because of its wide range of applications, especially in the biomedical field [1]. HA is a naturally occurring mineral found in rocks and is a major constituent of teeth and bones [2,3]. HA, which constitutes about 70% of human bone by mass, has gained critical importance in bone tissue engineering applications due to its similar crystallography with minerals of natural bones, bioactivity and non-toxicity, which are essential properties of a material suitable for bone regeneration [4]. Crystalline HA is present at the nanometre length scale within the bone hierarchy and along with collagen fibrils and extrafibrillar matrix of non-collagenous proteins assemble into the lamellar structure of bone. Even though autologous grafts provide the desired characteristics, they are also associated with some drawbacks, such as the donor site morbidity, primitive injuries and high cost. The main disadvantage associated with allografts is the dearth of osteogenic characteristics, harvesting and preservation [5]. The biomedical applications of synthetic materials include exceptional bioactivity, cellular responsiveness, entrapment and meticulous release of biologically active molecules. The tunable surface chemistry during synthesis opens up new horizons towards specific cellular targeted applications [6]. As a result HA finds wide range of critical applications in drug delivery [7], tooth enamel repairing [8], filler for bone repair [9], injectable bone cement [10] and as a coating for prosthetic implantation [11]. When HA is mixed with other phosphates, such as β-tricalcium phosphate (β-TCP) and calcium pyrophosphate, it promotes cell adhesion and osteogenic characteristics [12]. Bone contains about 70% calcium phosphate by weight, which makes it an important material especially for hard tissue development. Bone has a fairly complex, hierarchical [13], three-dimensional structure encompassing self-assembled discrete building blocks composed of collagen triple helices, mineralized by bioapatite nanocrystals [14,15], which is formed by a cell-mediated process.

Calcium phosphates and HA can be synthesized through a variety of well-developed techniques such as wet-chemical precipitation [16], solid-state reaction [17], reverse micro-emulsion [18], chemical vapour deposition [19] and sol–gel process. Sol–gel process is well known for its flexibility and offers different chemistries to fabricate a wide range of structural materials with particle sizes ranging from nanometre to micrometre scale [20,21]. Owing to the considerable osteogenic properties of HA [22], it has been incorporated into different natural and synthetic polymeric systems to produce nanocomposites with improved mechanical and biomedical properties [23–25]. Polymeric materials widely used as matrices for such nanocomposites include chitosan [26], collagen [27], gelatin [28], polycaprolactone [29] and poly(lactic acid) [30]. Effects of different parameters such as the reaction temperature and ageing time on the morphology and crystallinity of synthetic calcium phosphate powders have been extensively investigated. Increase in overall crystallinity and crystal size were observed with temperature and ripening time. Increase in crystallinity was reported to produce particles with regular and smooth surfaces [31]. As expected a change in Ca/P mole ratio strongly influenced the product formed where a decrease in Ca/P ratio resulted in an increase in the amount of β-TCP formed [32]. David *et al*. studied the effect of post synthesis thermal treatment on biphasic mixture of HA and β-TCP. They observed that after heat treatment at 900°C, biphasic calcium phosphate particles were homogeneous and their micro-pore density was much improved [33]. Post synthesis calcination also increased the particle size and crystallinity [34]. Similarly, Jung *et al*. synthesized HA through spray pyrolysis by using high temperature flame and examined the effect of solvent on physical properties of particles obtained. They used water and ethanol/water mixture (1/2 v/v) and observed that larger size and bimodal particles were formed through water only, while monodisperse and nano-sized particles were produced when ethanol/water mixture was used [35].

In this study, we explored the effects of various solvent parameters such as dielectric constant and polarity on the structure and the properties of calcium phosphates synthesized. For this purpose, distilled water, tetrahydrofuran (THF) and *N*,*N*-dimethylformamide (DMF) were chosen as the reaction solvents to synthesize calcium phosphate through sol–gel process. Physical and chemical properties of the calcium phosphate powders prepared were analysed by various characterization techniques, such as Fourier transform infrared (FTIR) and Raman spectroscopy, scanning electron microscopy (SEM), X-ray fluorescence (XRF) and X-ray diffraction (XRD).

## 2. Material and methods

### 2.1. Materials

Reagent-grade diammonium hydrogen phosphate ((NH_4_)_2_HPO_4_; DAHP), calcium nitrate tetrahydrate (Ca(NO_3_)_2_·4H_2_O; CNTH), THF, DMF and ammonia solution (28–30%; NH_4_OH) were obtained from Merck and used as received without further purification. Triple distilled water was used for all experiments. Dielectric constants (*ε*) of THF, DMF and water are 7.58, 36.7 and 80.1, respectively.

### 2.2. Methods

#### 2.2.1. Calcium phosphate synthesis

**Figure 1.**
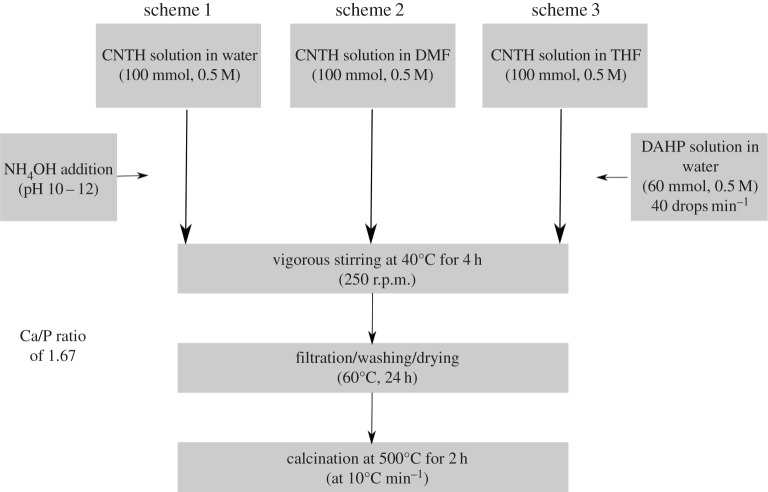
Schematic of the reaction schemes followed in calcium phosphate synthesis through sol–gel process.

Calcium phosphate synthesis was carried out using a sol–gel method reported earlier [36,37], with minor variations. Three different synthetic procedures were followed as presented in figure 1. DAHP was dissolved in distilled water in all reaction schemes, while CNTH was dissolved in distilled water (figure 1, scheme 1), THF (scheme 2) and DMF (scheme 3). All other reaction parameters such as temperature, time, addition rate, washing cycles, drying time and temperature were kept the same in all synthetic processes used.

The pH of aqueous solutions was adjusted in the range of 10–12 by the dropwise addition of NH_4_OH solution. For mixed (organic/water) solvent systems, NH_4_OH was added into 5 ml of water and then diluted with the organic solvent. CNTH (100 mmol) was dissolved in water, THF or DMF while DAHP (60 mmol) was dissolved in water to reach the final concentration of 0.5 M each. CNTH solution was introduced into a 500 ml three-neck round bottom Pyrex flask, equipped with an overhead stirrer, addition funnel and thermometer. DAHP solution was added with an addition rate of approximately 40 drops min^−1^ through an addition funnel while the mixture was vigorously stirred at 250 r.p.m. Water bath was used to maintain the temperature at 40 ± 2°C. After complete addition of DAHP solution, reaction was continued for additional 4 h. The products obtained were filtered through a filter paper (Filter-lab 1525), washed several times with cold distilled water and then dried in an air oven at 60°C for 24 h. All powders obtained were calcined in a furnace at 500°C for 2 h, which was heated with a heating rate of 10^°^C min^−1^. After calcination, the powders were directly used for characterization.

#### 2.2.2. Characterization techniques

Attenuated total reflectance (ATR) FTIR spectra were acquired on a Thermo Scientific iS50 FTIR with ATR attachment. Absorption spectra were collected by obtaining 64 scans at autogain with a resolution of 2 cm^−1^. X-ray diffractograms were obtained using a Bruker D8 Discover XRD system. Cu-K*α* X-ray generator with a wavelength of 1.54184 Å at a voltage 40 kV and 40 mA current was used. Surface and morphological analyses were conducted using a Zeiss Ultra Plus field emission scanning electron microscope (FESEM) equipped with an energy dispersive X-ray detector for elemental analysis. Powder sample was dispersed in formic acid to obtain a 0.1% (w/w) suspension, which was spin coated on silicon wafer to make the surface fairly conductive without using gold/carbon sputtering. Secondary electron (SE) detector images were obtained to analyse the morphology of the nanoparticles obtained. Raman analysis was performed on a Renishaw Invia-Raman microscope by using either 532 or 785 nm laser sources. Ca/P atomic ratio was determined by XRF using a Bruker Tiger S8 instrument.

## 3. Results and discussion

### 3.1. X-ray diffraction analysis

**Figure 2.**
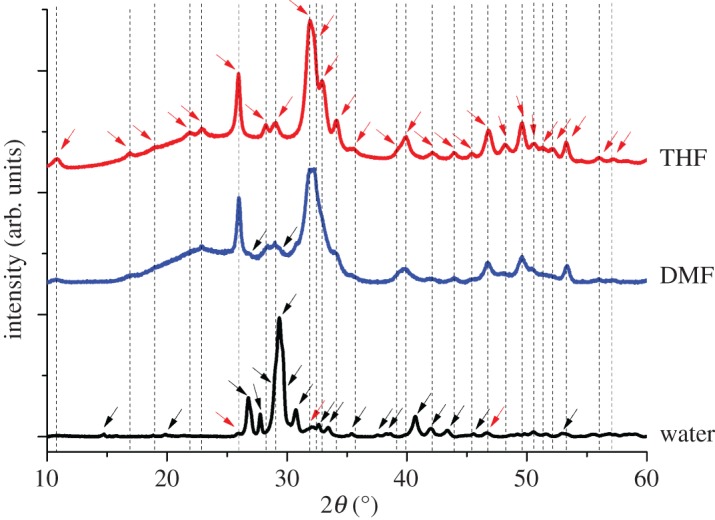
XRD analysis of calcined calcium phosphate powders synthesized by using water, THF and DMF as the reaction co-solvent.

Crystallographic structures of calcium phosphate powders synthesized were determined by wide angle XRD analysis. Diffractograms for different samples are reproduced in figure 2, where characteristic peaks for HA are shown with red arrows and peaks for β-calcium pyrophosphate (β-CPF) are marked with black arrows at their corresponding 2*θ* angles.

XRD patterns for all samples were analysed through DIFFRACT.SUITE EVA v.4.2. Samples were compared with database, matched with cards for HA (PDF-04-014-8416) and β-CPF (PDF-00-009-0346), and diffractograms are provided in the electronic supplementary material. Diffraction peaks for HA and β-CPF crystal planes along with *h*, *k*, *l* indices, *d* spacing value and their respective angles are tabulated in table 1.

**Table 1.** XRD analysis of HA and β-CPF.

hydroxyapatite (Ca_5_(PO_4_)_3_(OH))			β-calcium pyrophosphate (β-Ca_2_P_2_O_7_)		
angle 2*θ* (degrees)	*d* value (Å)	*h*, *k*, *l*	angle 2*θ* (degrees)	*d* value (Å)	*h*, *k*, *l*
10.85	8.15	1,0,0	14.68	6.03	0,0,4
16.89	5.25	1,0,1	19.80	4.48	1,0,4
18.84	4.71	1,1,0	26.91	3.31	2,0,1
21.79	4.08	2,0,0	27.68	3.22	2,0,2
25.98	3.43	0,0,2	28.87	3.09	2,0,3
28.23	3.16	1,0,2	29.56	3.02	0,0,8
28.96	3.08	2,1,0	29.87	2.99	2,1,0
31.82	2.81	2,1,1	30.78	2.90	2,1,2
32.29	2.77	1,1,2	32.55	2.75	2,0,5
32.94	2.72	3,0,0	33.41	2.68	2,1,4
34.16	2.62	2,0,2	35.27	2.54	2,1,5
35.52	2.53	3,0,1	37.41	2.40	2,1,6
39.29	2.29	1,2,2	38.52	2.34	1,1,9
39.84	2.26	1,3,0	40.61	2.22	3,0,1
42.05	2.15	1,3,1	42.01	2.15	3,0,3
44.03	2.06	1,1,3	43.19	2.09	3,0,4
45.48	1.99	2,0,3	45.47	1.99	1,1,11
46.80	1.94	2,2,2	52.78	1.62	4,1,1
50.55	1.80	3,2,1			
51.33	1.78	1,4,0			
52.18	1.75	4,0,2			
53.44	1.71	0,0,4			
55.98	1.64	3,2,2			
57.29	1.61	1,3,3			

### 3.2. Attenuated total reflectance Fourier transform infrared analysis

**Figure 3.**
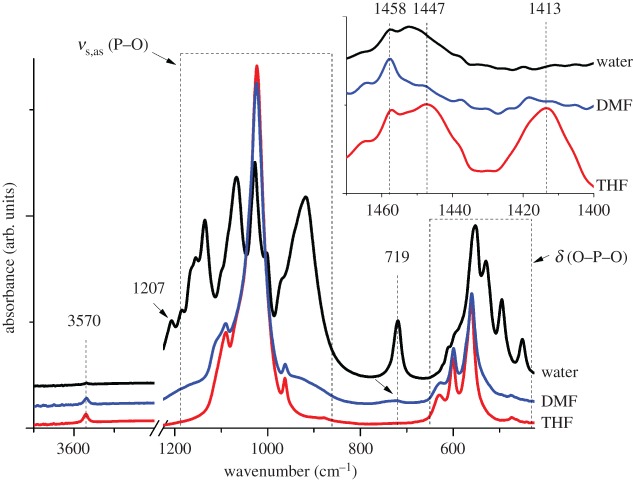
ATR-FTIR analysis of calcium phosphate powders obtained by using different reaction solvent systems.

To identify the characteristic functional groups in the powders synthesized, FTIR spectroscopy was used. The spectra obtained for the products synthesized in water, THF and DMF solvent systems are provided in figure 3.

As can clearly be seen in figure 3, strong symmetrical and asymmetrical $\text{P}{{\text{O}}_{4}}^{3-}$ stretching modes characterized by complex bands in the region of 1200–900 cm^−1^, which are typically associated with HA, are observed for all products. Fairly small but well-defined hydrogen-bonded OH stretching band appears at 3570 cm^−1^, which is more obvious in THF- and DMF-based products due to the presence of HA [Ca_10_(PO_4_)_6_(OH)_2_] and less observable in water-based product, which is mainly composed of β-CPF [β-Ca_2_P_2_O_7_]. Bending vibrations for O–P–O appear in the range of 650–425 cm^−1^. There are two more absorption bands appearing at 1207 and 719 cm^−1^, which correspond to pyrophosphate (${\text{P}}_{2}{{\text{O}}_{7}}^{4-}$) stretching modes [36]. These bands are observed significantly only in the water-based synthesis due to the large presence of β-CPF phase. Very weak signal at 719 cm^−1^ as indicated by black arrow head was present in DMF-based synthesis.

Additionally, various other absorption bands were also observed in the range of 1470–1400 cm^−1^ as can be seen in the inset in figure 3. These bands appearing at 1413, 1447 and 1458 cm^−1^ can be assigned to $\text{C}{{\text{O}}_{3}}^{2-}$ [38,39]. This is probably due to the absorption of carbon dioxide from atmosphere, which interacts with nano-HA powders. THF-based system shows more carbonate presence than others because hydroxides have very high affinity to carbonate, especially at high pH values. This suggests that some tracing amounts of $\text{P}{{\text{O}}_{4}}^{3-}$ have been substituted by carbonate [22]. Ambient CO_2_ dissolved can be limited by either performing the reaction under nitrogen atmosphere or sintering the synthesized powder at elevated temperature. Replacement of $\text{C}{{\text{O}}_{3}}^{2-}$ with $\text{P}{{\text{O}}_{4}}^{3-}$ enhances HA dissolution and results in the reduction of the crystallite size [40].

#### 3.2.1. Raman analysis

**Figure 4.**
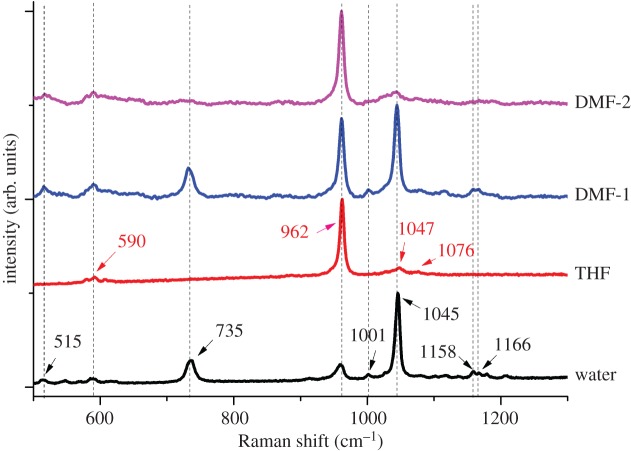
Raman analysis of calcium phosphate powders obtained from different reaction schemes.

**Figure 5.**
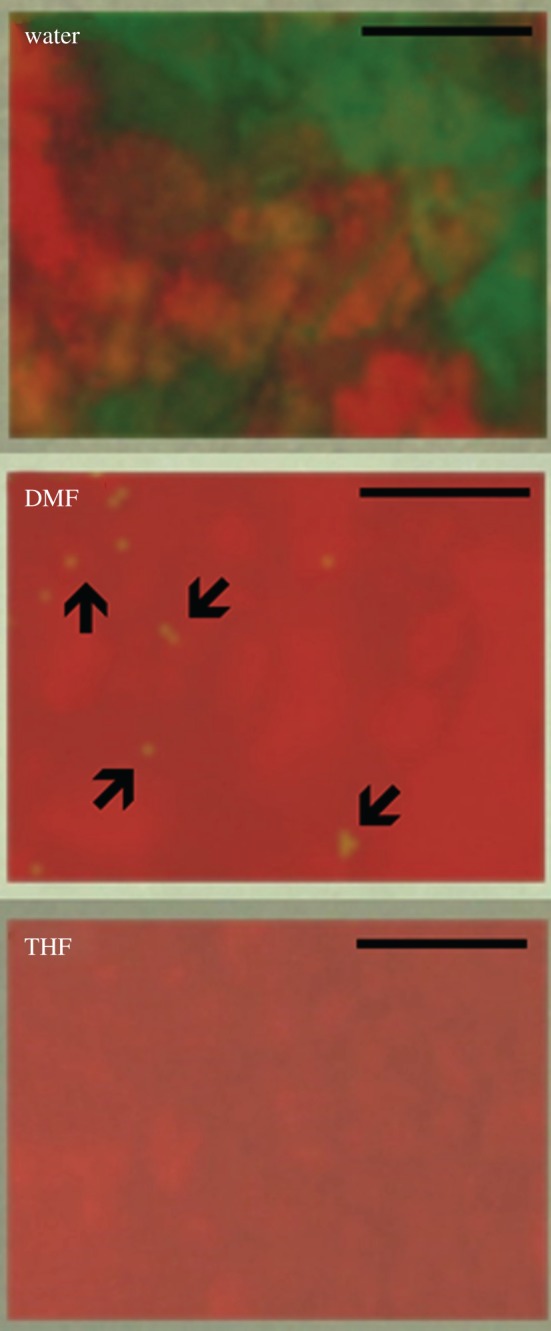
Raman mapping of synthesized calcium phosphate phases (scale bar, 20 µm): HA (red), β-CPF (green).

Owing to the differences in the bonding structures of various calcium phosphates present in the powders produced by using different solvent systems, we used Raman spectroscopy to distinguish them from each other. Raman spot analysis and Raman mapping were performed for all samples. Raman spectra are provided in figure 4, whereas the Raman mapping data are provided in figure 5. Raman spectra for powders synthesized in THF and water were obtained by using solid-state laser of 532 nm wavelength having beam size of 1.5–2 µm in diameter, while for DMF-based samples analysis was done with line laser (785 nm) of dimensions 1.5–2 × 20 µm. As DMF-based system has more mixed phases, we also accordingly analysed it through the line laser to get signals from the minor phase of β-CPF.

Raman peaks for β-CPF appear at 515, 735, 1001, 1158, 1166 and the most dominating peak at 1045 cm^−1^ as indicated by black arrow heads in figure 4, while for HA, peaks appear at 590, 1047, 1076 and the major peak at 962 cm^−1^ [3,41]. Sample prepared in THF displayed a single major Raman absorption peak centred at 962 cm^−1^ and three very small peaks at 590, 1047 and 1076 cm^−1^, clearly indicating the formation of HA only. On the other hand, the major peak for the sample prepared in water was at 1045 cm^−1^, with smaller absorption peaks at 735 and 962 cm^−1^ and fairly small peaks at 515, 1158 and 1166 cm^−1^. These spectral data demonstrate that the major product formed in aqueous medium is β-CPF, with minor amount of HA. In DMF-based samples, analyses were performed at two different points that yielded different spectra indicating the presence of a minor amount of β-CPF phase mixed with the bulk HA phase. In DMF-2 spectrum (figure 4), only peaks for HA phase were observed, whereas for another spot analysis (DMF-1 spectrum), peaks for both HA and β-CPF were observed. Results of Raman spectral analyses confirm that THF-based synthesis resulted in only HA phase formation, water-based synthesis yielded larger phase of β-CPF and a minor phase of HA while DMF-based synthesis generated larger phase of HA and a minor phase of β-CPF.

Raman spot analysis and mapping is a powerful tool for checking the purity of the materials synthesized by technically selecting appropriate irradiated laser type and analysis parameters. Even if a material is in the powder form and it is ‘XRD amorphous’ (i.e. does not produce distinct XRD peaks) it can still have strong peaks in a Raman spectrum because Raman scattering is sensitive to ordering within atomic clusters while XRD is at larger scale [3]. As shown in figure 5, Raman mapping was also performed using 532 nm laser to show the presence of different phases in the samples. Red colour shows the presence of HA and green colour represents the β-CPF phase in the calcium phosphate mixture. In water-based synthesis, we can clearly see the mixture of HA and β-CPF, in DMF-based synthesis, a fairly minor phase of β-CPF is present as indicated by the black arrows, whereas the product obtained in THF-based synthesis consists of purely HA phase. These results strongly confirm our hypothesis that phase of synthesized calcium phosphate depends on the nature and properties of the solvent media, when other processing conditions (such as temperature, time) are kept constant.

#### 3.2.2. Surface analysis

**Figure 6.**
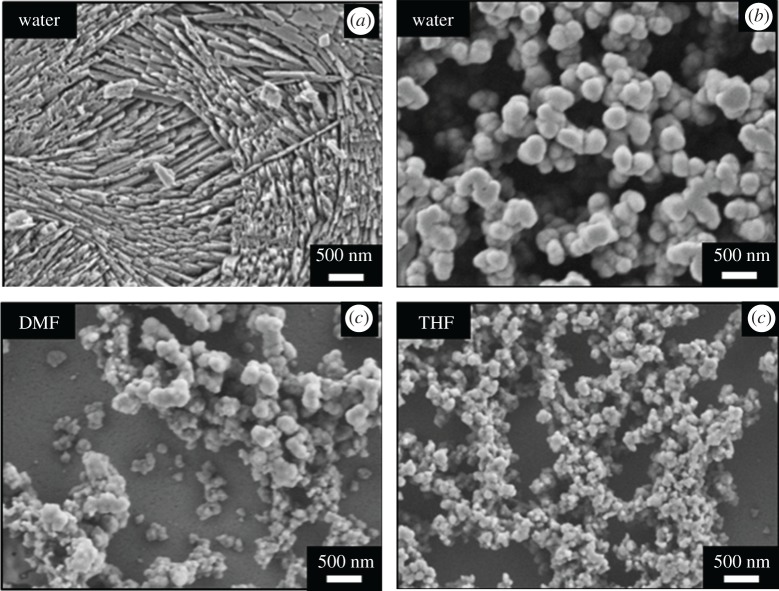
FESEM images of calcium phosphate powders synthesized in different solvent media.

SE images of the synthesized calcium phosphate powders were collected using FESEM. All three powders were dispersed in formic acid at a concentration of 0.1 wt% and spin coated at 1000 r.p.m. for 70 s on cleaned silicon wafers. After completely evaporating the solvent in a vacuum oven, samples were analysed at accelerating voltage of 2 kV without gold/carbon sputtering. Acquired SE images are provided in figure 6.

As can clearly be seen in figure 6*a*,*b*, in water-based synthesis, formation of biphasic calcium phosphate showing different morphologies was observed, where plate-like structure corresponds to β-CPF (figure 6*a*). HA was observed as larger, spherical particles with fairly uniform sizes when compared to other samples (figure 6*b*). Higher amount of β-CPF was found to be present in the water-based sample as compared to HA. In DMF-based synthesis, only spherical particles were observed in the SEM images (figure 6*c*) corresponding to HA particles; however, a broader particle size distribution was also observed. In THF-based synthesis spherical HA particles with fairly homogeneous size distribution were observed in SEM (figure 6*d*). These SEM results strongly support the results obtained from FTIR, XRD and Raman studies, clearly showing the formation of fairly pure HA in THF solution, while the water-based synthesis yields large amounts of β-CPF and minor amount of HA. DMF-based product comprises mainly HA with a small amount of β-CPF.

#### 3.2.3. Effect of solvent on physical and chemical properties

As we have demonstrated, the dielectric constant and polarity of the reaction solvent have a critical effect on the nature of the product obtained during the synthesis of calcium phosphate through sol–gel process. Solvent can influence the reaction rate and hydrolysis thus ultimately the structure of the synthesized material [42,43]. Dielectric constant of a solvent, which is related to the strength of its intermolecular forces and polarity, determines the efficiency of the solvent in separating the electrolytes into ions. Solvents with high dielectric constants favour complete dissociation of electrolytes, while solvents with low dielectric constant cause ion pairing. Dielectric constant of solvent mixture can be calculated as a simple additive function of weighted average of the dielectric constants of the components [44,45]. Calculated dielectric constant values for all three reaction schemes are given in table 2. As Ca/P ratio for β-CPF and HA is 1 and 1.67, respectively, keeping all other parameters constant, we believe that as the polarity of the reaction medium increases, reaction rate also increases, resulting in the formation of higher amounts of β-CPF rather than HA. Hence, concentration of individual phase in a multiphasic calcium phosphate synthesis can be monitored by carefully controlling the polarity and the dielectric constant of the solvent mixture. Surface area (*S*_BET_) and average particle sizes were determined by N_2_ adsorption isotherms according to BET (Brunauer–Emmett–Teller) procedure and are provided in table 2. Results obtained are in good agreement with FESEM analysis. Water-based synthesis yields a powder with large plate-like structure for β-CPF as compared to smaller spherical shaped HA particles in the other two solvents systems. Average particle size is very high when compared to the others and hence very low surface area. Average particle size in THF-based synthesis is slightly larger than DMF-based system due to low rate of reaction [46]. Results obtained in THF- and DMF-based synthesis are almost comparable.

**Table 2.** Influence of the dielectric constant of the solvent mixture on BET, XRF and XRD analysis of calcium phosphate synthesized.

		water-based	DMF-based	THF-based
		synthesis	synthesis	synthesis
dielectric constant of solvent mixture		78.54	54.28	37.4
BET analysis	average particle size, *d*_BET_ (nm)	247	73	80
	surface area, *S*_BET_ (m^2 ^g^−1^)	24	82	75
XRF analysis	Ca/P ratio	1.07	1.45	1.78
XRD analysis	crystallite size (nm)	19.4^a^	27.0^b^	25.2^b^
	crystallinity (%)	70.7	25.9	31.9

^a^*k*-value of 0.89.

^b^*k*-value of 1.

Elemental analysis of the products obtained was conducted using XRF and calculated Ca/P values are provided in table 2. In water-based synthesis, the ratio is quite low, 1.07, because of the presence of larger β-CPF phase with a Ca/P molar ratio of 1. DMF-based synthesis has a much higher Ca/P ratio of 1.45, where HA is the major and β-CPF is the minor product. THF-based synthesis yielded the maximum value of Ca/P ratio of 1.78, clearly indicating the formation of pure HA. Slightly higher Ca/P value of this system compared to the theoretical Ca/P value of 1.67 for pure HA phase can be due to the replacement of the phosphate groups with carbonates, as discussed in the ATR-FTIR section.

Broadening of the X-ray reflection peaks can be used to estimate the crystallite size in a direction vertical to the crystallographic phase by using Debye--Scherrer equation [47]

3.1 $$D=\frac{k\lambda }{\beta_{1/2}\;\text{cos}\;\theta },$$

where *D* is the size in Å, *k* the shape factor and its value was used as 1 for THF- and DMF-based synthesized powder and 0.89 for water-based powder due to the morphology difference. *λ* is the wavelength used, which is 1.54184 Å, *θ* is the diffraction angle and *β*_1/2_ is the selected diffraction peak width at half height expressed in radians. (002) and (201) peaks were selected for HA and β-CPF crystal size calculation because of their prominent and distinct nature. Crystallite size and per cent crystallinity for each synthesized powder were calculated/estimated using Diffract Eva® software and results are given in table 2. Relatively smaller crystallite size and highest amount of crystallinity were observed in water-based calcium phosphate powder due to the presence of a fairly large β-CPF phase in the mixture. The reaction rate was highest among all reaction schemes due to the highest polarity and dielectric constant of the system that ultimately led to calcium pyrophosphate crystal growth instead of HA.

## 4. Conclusion

In this study, critical influence of the dielectric constant and polarity of the reaction solvent on calcium phosphate synthesis was explored. Main aim of the study was the investigation of the solvent effect on the synthesis of HA with a Ca/P ratio of 1.67 through a sol–gel process. This was achieved by reacting ‘Ca’ and ‘P’ precursors in different solvent mixtures, where pure water, water/DMF and water/THF mixtures with different polarities and dielectric constants were used as the reaction media. After washing, drying and calcination of the synthesized powder, physical and chemical characterization was performed by FESEM, BET, ATR-FTIR, Raman, XRF and XRD techniques. Water-based reaction medium with highest dielectric constant yielded mainly β-CPF with a minor amount of HA. In DMF/water-based synthesis, HA was obtained as the major phase with a very minor amount of β-CPF. In THF/water solvent system with the lowest dielectric constant, pure HA was synthesized. Our results clearly show the significant effect of the dielectric constant and polarity of the reaction medium on calcium phosphate synthesis through sol–gel process, while keeping the other factors constant. By carefully controlling the solvent parameters, one can control the amount of different calcium phosphate phases produced, which may play critical roles for specific cellular activities and targeted applications.

## Supplementary Material

Supplementary Data

## Data accessibility

Data supporting this study have been uploaded as the electronic supplementary material and can be found here [48] (http://dx.doi.org/10.5061/dryad.6fj3v).
