# Supplementary material for: Effect of reaction solvent on hydroxyapatite synthesis in sol–gel process
Source: R Soc Open Sci. 2017 Dec 20;4(12):171098. doi: 10.1098/rsos.171098 (PMC5750015; doi:10.1098/rsos.171098)
Supplement: Supplementary Data [file rsos171098supp1.docx]

**Supplementary Data**


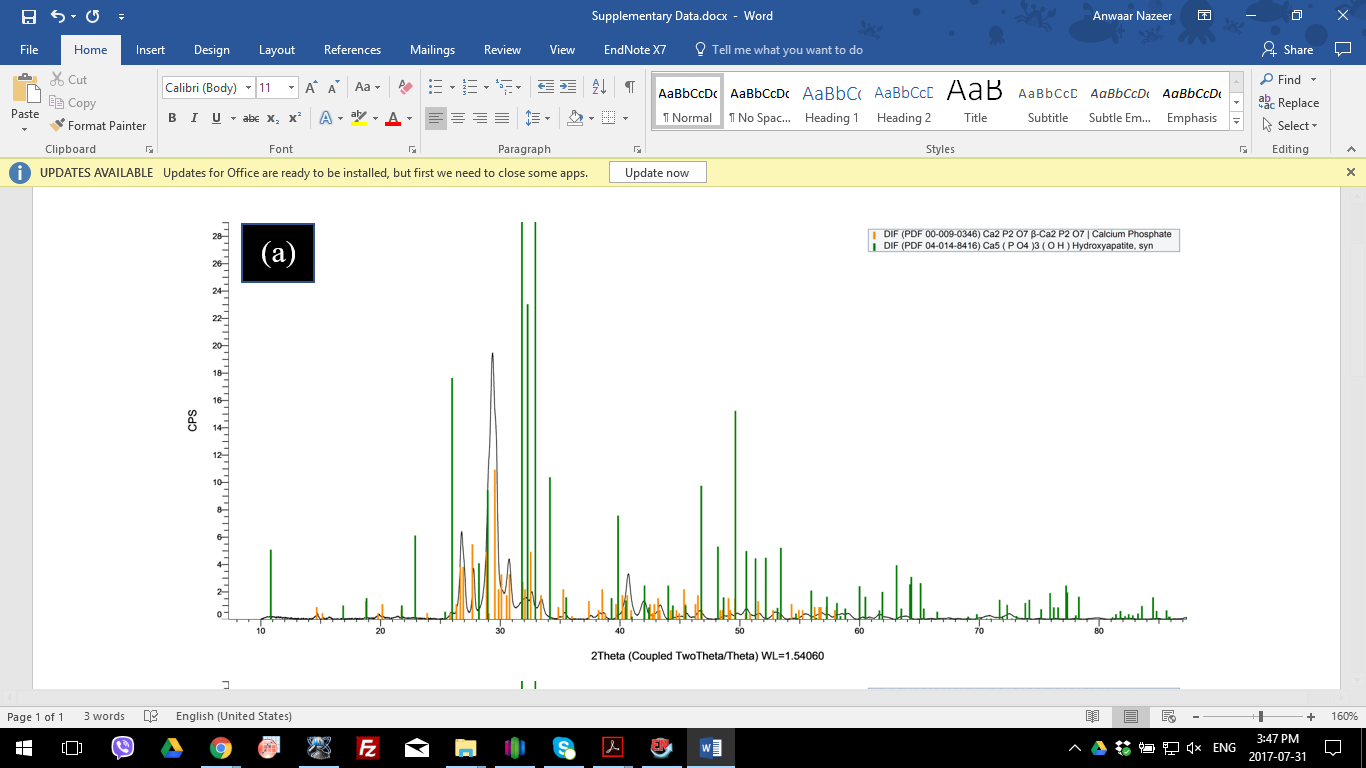


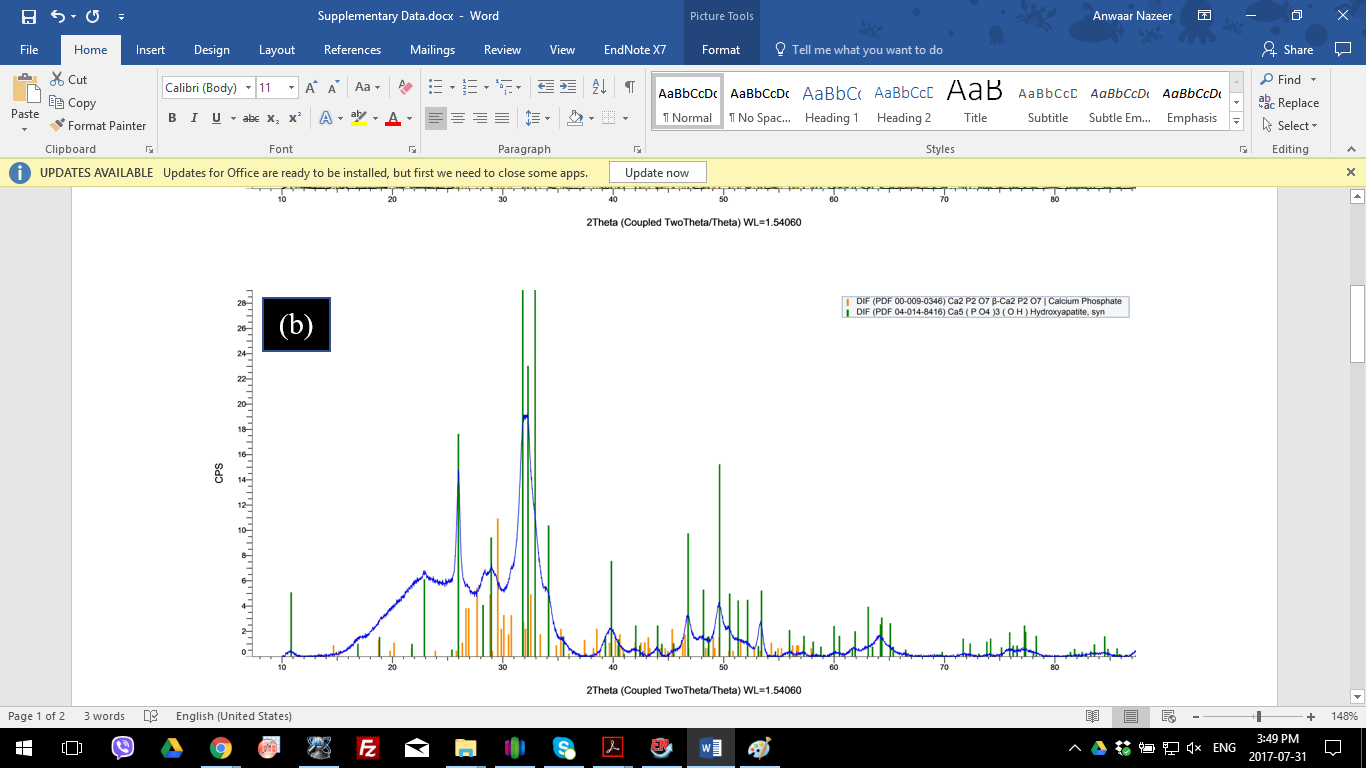


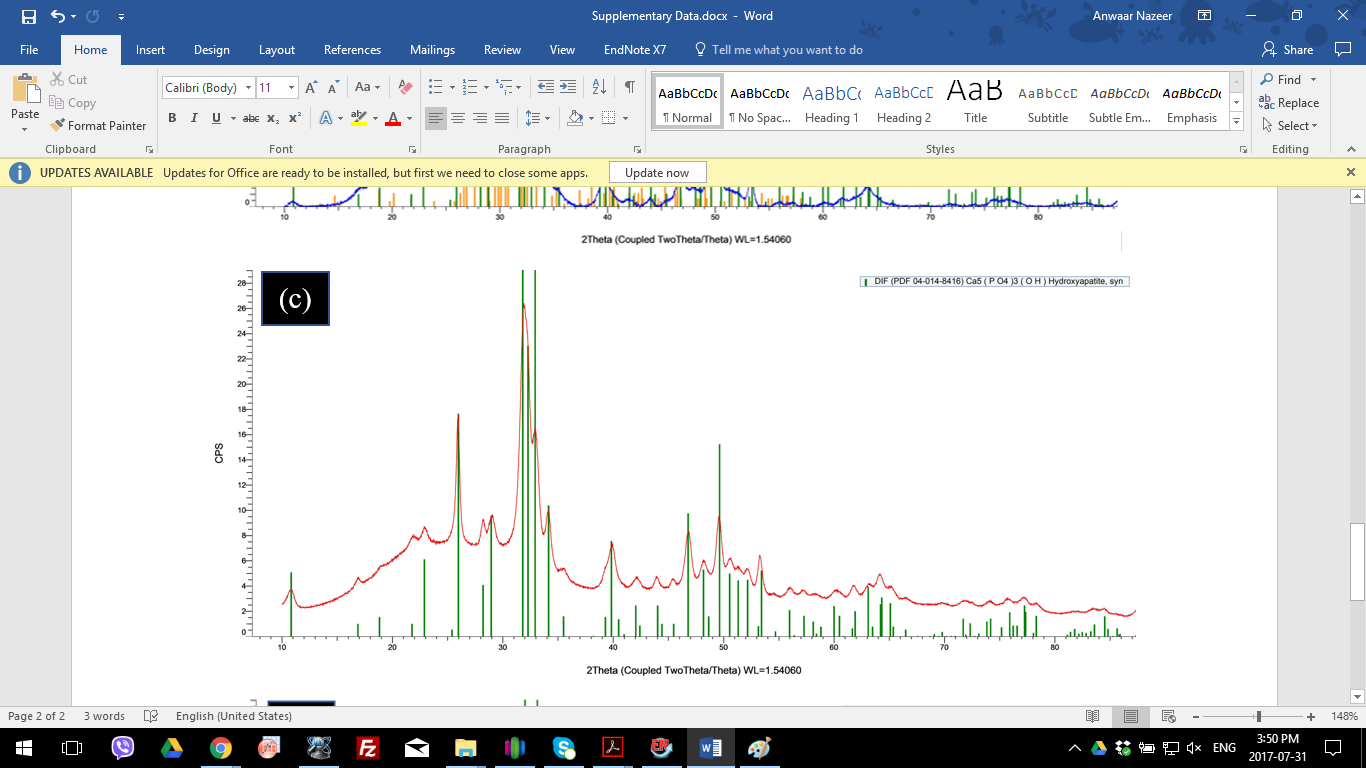


Figure 1: XRD diffractograms of (a) Water, (b) DMF and (c) THF based synthesis with their respective database
